# The Novel Regulatory Role of the lncRNA–miRNA–mRNA Axis in Chronic Inflammatory Airway Diseases

**DOI:** 10.3389/fmolb.2022.927549

**Published:** 2022-06-13

**Authors:** Xin Qiao, Gang Hou, Yu-Lin He, Dong-Fang Song, Yi An, Abdullah Altawil, Xiao-Ming Zhou, Qiu-Yue Wang, Jian Kang, Yan Yin

**Affiliations:** ^1^ Department of Pulmonary and Critical Care Medicine, First Affiliated Hospital of China Medical University, Shenyang, China; ^2^ Department of Pulmonary and Critical Care Medicine, Center of Respiratory Medicine, China-Japan Friendship Hospital, Beijing, China; ^3^ Respiratory Department, Center for Pulmonary Vascular Diseases, Fuwai Hospital, National Center for Cardiovascular Diseases, Chinese Academy of Medical Sciences, Peking Union Medical College, Beijing, China

**Keywords:** long noncoding RNAs, miRNAs, mRNAs, asthma, COPD

## Abstract

Chronic inflammatory airway diseases, characterized by airway inflammation and airway remodelling, are increasing as a cause of morbidity and mortality for all age groups and races across the world. The underlying molecular mechanisms involved in chronic inflammatory airway diseases have not been fully explored. MicroRNAs (miRNAs) and long noncoding RNAs (lncRNAs) have recently attracted much attention for their roles in the regulation of a variety of biological processes. A number of studies have confirmed that both lncRNAs and miRNAs can regulate the initiation and progression of chronic airway diseases by targeting mRNAs and regulating different cellular processes, such as proliferation, apoptosis, inflammation, migration, and epithelial–mesenchymal transition (EMT). Recently, accumulative evidence has shown that the novel regulatory mechanism underlying the interaction among lncRNAs, miRNAs and messenger RNAs (mRNAs) plays a critical role in the pathophysiological processes of chronic inflammatory airway diseases. In this review, we comprehensively summarized the regulatory roles of the lncRNA–miRNA–mRNA network in different cell types and their potential roles as biomarkers, indicators of comorbidities or therapeutic targets for chronic inflammatory airway diseases, particularly chronic obstructive pulmonary disease (COPD) and asthma.

## Introduction

Chronic inflammatory airway diseases, including chronic obstructive pulmonary disease (COPD), bronchial asthma, bronchiectasis and cystic fibrosis (CF), remain a major problem worldwide (Soriano et al., 2020). Asthma and COPD both cause airway obstruction due to cellular and structural changes, known as airway remodelling (Aoshiba and Nagai, 2004; Hogg, 2004). Angiogenesis initiates the remodelling of the bronchial vasculature that eventually contributes to the progression of asthma and COPD (Global Strategy for Asthma Management and Prevention, 2012; Hislop, 2002; Global Initiative for Chronic Obstructive Lung Disease (GOLD), 2013). Bronchiectasis is characterized by irreversible bronchial dilatation with associated chronic cough, mucus hypersecretion, and recurrent pulmonary infections (Polverino et al., 2017). CF, a major disorder associated with bronchiectasis, is caused by mutation of a gene called cystic fibrosis transmembrane conductance regulator (CFTR), and its dysfunction results in mucus retention and chronic infection with subsequent local airway inflammation (Elborn, 2016). They are increasing as a cause of morbidity and mortality with a serious disease burden across the world. The molecular mechanisms of the pathophysiological processes involved in these diseases have not been completely interpreted. The identification of the pivotal molecules involved in chronic inflammatory airway diseases is urgently needed for exploring effective prevention measures and therapeutic strategies. In recent decades, noncoding RNAs (ncRNAs), characterized by the absence of significant protein‐coding potential, have been comprehensively investigated. As two major members of the ncRNA family, long noncoding RNAs (lncRNAs) and microRNAs (miRNAs) play key roles in regulating cell differentiation (Liang and Tang, 2020), proliferation (Wang et al., 2021a), autophagy (Li et al., 2021), and apoptosis (Wang et al., 2020). LncRNAs, a novel class of RNA transcripts with a length greater than 200 nucleotides, have been thought to have no biological function due to their lower expression levels compared with protein‐coding genes, but growing evidence has confirmed that lncRNAs can regulate target gene expression from the transcriptional and posttranscriptional to posttranslational levels and play critical roles in multiple biological processes (Ransohoff et al., 2018). Unlike lncRNAs, miRNAs are a family of evolutionarily conserved ncRNAs and can bind to the 3′‐untranslated region (3′‐UTR) of messenger RNAs (mRNAs), leading to mRNA translation inhibition or mRNA degradation, which play a significant role in regulating cellular bioactivity (Li et al., 2022; Smith et al., 2022). Recently, a variety of studies have confirmed that noncoding RNAs (ncRNAs) participate in the development of chronic inflammatory airway diseases (Li et al., 2019a). Moreover, a novel regulatory mechanism among lncRNAs, miRNAs and their mRNA targets has been found in chronic inflammatory airway disease. For example, lncRNA bromodomain adjacent to zinc finger domain protein 2B (lnc-BAZ2B) promoted pulmonary inflammation and mucus secretion via BAZ2B/interferon regulatory factor 4 (IRF4) *in vitro* (Xia et al., 2021). The overexpression of miR-145-5p attenuated cigarette smoking extract (CSE)-stimulated apoptosis and inflammation in human bronchial epithelial cells (Li et al., 2019b). LncRNA cancer susceptibility candidate 7 (CASC7) promoted the phosphorylation of glucocorticoid receptors to increase the sensitivity of glucocorticoids in patients with severe asthma by targeting the miR-21/phosphoinositide 3-kinase (PI3K)/protein kinase B (Akt) signalling pathway. This review summarizes the current progress of research on the interaction between lncRNAs and miRNAs to provide insight into their potential as new biomarkers or therapeutic targets of chronic inflammatory airway diseases.

## NcRNAs in Chronic Inflammatory Airway Diseases

NcRNAs are commonly classified into two categories, housekeeping and regulatory ncRNAs, according to their regulatory roles. MiRNAs (transcripts between 19 and 25 nucleotides) and lncRNAs (transcripts >200 nucleotides) are major members of the regulatory ncRNA family. MiRNAs negatively regulate gene expression by target mRNA cleavage and degradation or translation inhibition, while lncRNAs negatively or positively regulate various stages of gene expression through interactions with DNA, RNA or proteins (ChewChew et al., 2018). Currently, ncRNAs are discovered by broad-range profiling methods, including bioinformatic analysis, microarrays, and next-generation sequencing, which could measure the differential expression of thousands of ncRNAs (Vencken et al., 2015; Beermann et al., 2016). Validation of the screening results can be done with low effort and relatively low costs by quantitative real-time PCR (qRT-PCR) or western blot. Human samples such as blood, urine, sputum, and lung tissues have been shown to exhibit a differentially expressed ncRNAs (Liang et al., 2019; Jakwerth et al., 2021; McDonough, et al, 2019), underscoring their potential to serve as biomarkers. In terms of the bio-sample, the blood might be better for investigating ncRNA as biomarkers in chronic inflammatory airway diseases since inflammatory processes from the lung could spill into the blood, and ncRNA obtained from blood could be representative of the inflammatory response in the lung and other tissues. In addition, it may be performed more easily than invasive methods such as lung biopsies, bronchial brushings or bronchoalveolar lavage fluid.

Emerging evidence suggests that ncRNAs account for the pathophysiological alteration of chronic inflammatory airway diseases. Several miRNAs are associated with the pathophysiology of COPD. For example, miR-183 expression was increased in COPD patients and could regulate Ca^2+^-activated K^+^ channels β1 subunit (BKCaβ1) expression related to the severity of COPD (Szymczak et al., 2016). Zhong et al. (Zhong et al., 2019) identified a significant upregulation of hsa-miR-664a-3p in COPD patients, and its target gene four and a half LIM domains 1 (FHL1) was downregulated and positively correlated with forced expiratory volume in 1s (FEV_1_)/forced vital capacity (FVC)%. Savarimuthu et al. (Savarimuthu Francis et al., 2014) found that miR-34c levels were related to the severity of emphysema in COPD patients by regulating serine protease inhibitor clade E member 1 (SERPINE1). Furthermore, the upregulated miR-223 (Leuenberger et al., 2016) and downregulated miR-149-3p (Shen et al., 2017), miR-145-5p (Dang et al., 2019), and miR-29b (Li et al., 2019b) acted on histone deacetylase 2 (HDAC2), CCAAT/enhancer-binding protein beta (C/EBP-β), transforming growth factor beta1 (TGF-β1), Toll-like receptor 4 (TLR-4)/nuclear transcription factor-kappa B (NF-κB), kruppel-like factor 5 (KLF5), and bromodomain-containing protein 4 (BRD4), respectively, thereby regulating the inflammatory response in COPD patients. Several miRNAs are associated with the pathophysiology of asthma. For example, upregulation of miR-21 negatively regulates interleukin (IL)-12p35 (Lu et al., 2009), signal transducer and activator of transcription 4 (STAT4) (Wu et al., 2014), phosphatase and tensin homolog deleted on chromosome 10 (PTEN) (Liu et al., 2015), and HDAC2 (Kim et al., 2017), controlling airway hypersensitivity, Th1/Th2 balance, and airway smooth muscle cell proliferation and migration in different asthmatic models. The upregulated miR-943-3p (Shen et al., 2019) and downregulated miR-485 (Wang et al., 2018a) also target secreted frizzled-related protein 4 (SFRP4) and smad ubiquitin regulatory factor 2 (Smurf2), respectively, promoting proliferation of airway smooth muscle cells and leading to airway remodelling *in vivo* or *in vitro*. Li et al. (Li et al., 2020) found that the downregulation of miR-30a promoted fibrogenesis, autophagic flux, and airway remodelling by targeting autophagy related 5 (ATG5). Decreased expression of miR-181b-5p (Huo et al., 2016) and miR-221-3p (Zhang et al., 2018a) was reported in the epithelium of eosinophilic asthmatic patients, and their downregulation suppressed inflammatory cytokines (C-C motif chemokine ligand 11 (CCL-11, CCL-24, and CCL-26), suggesting the protective role of miR-181b-5p and miR-221-3p against airway eosinophilic inflammation. In addition, miRNAs are also involved in CF. For instance, miR-101-3p, miR-145-5p, miR-384, miR-494, and miR-600 directly target and regulate CFTR (Gillen et al., 2011; Oglesby et al., 2013; Dutta et al., 2019; Fabbri et al., 2021), while miRNA-138 (Ramachandran et al., 2012) indirectly regulates CFTR by targeting suppressor interacting 3A (SIN3A). The upregulated miR-155 (Bhattacharyya et al., 2011) and downregulated miR-199a-3p (Bardin et al., 2018), miR-17 (Oglesby et al., 2015), miR-93 (Fabbri et al., 2014) and miR-126 (Oglesby et al., 2010) promote IL-8 secretion, contributing to pulmonary inflammation in CF airways. Moreover, Huang et al. (Huang et al., 2019) found that differentially expressed miR-223-3p and miR-92b-5p were associated with PA colonization in bronchiectasis patients, and their expression levels were significantly correlated with sputum IL-β and IL-8 levels.

In addition to miRNAs, lncRNAs are also associated with the pathogenesis and development of chronic inflammatory airway diseases. For example, taurine-up-regulated gene 1 (TUG1) and metastasis-associated lung adenocarcinoma transcript 1 (Malat1) are upregulated in lung tissues of COPD patients, and their silencing inhibits the expression of fibronectin and α-smooth muscle actin (α-SMA) and improves the viability of human fetal lung fibroblast 1 (HFL1) cells following TGF-β pretreatment (Tang et al., 2016; Hu et al., 2020). HOXA-AS2 was downregulated in lung tissues from COPD patients, and the downregulation of HOXA cluster antisense RNA 2 (HOXA-AS2) suppresses CSE-induced human pulmonary microvascular endothelial cells (HPMECs) proliferation via Notch1 signalling *in vitro* (Zhou et al., 2020a). Furthermore, several lncRNAs are also associated with the regulation of airway inflammation and asthma. Zhang et al. (Zhang et al., 2016) found that brain cytoplasmic RNA 1 (BCYRN1) was upregulated in an asthma rat model and induced the proliferation and migration of airway smooth muscle cells (ASMCs) by targeting transient receptor potential canonical 1 (TrpC1). The upregulation of lncRNA transcription factor 7 (TCF7) (Fan et al., 2019) and lncRNA plasmacytoma variant translocation 1 (PVT1) (Austin et al., 2017) and their role in the regulation of ASMC proliferation and migration have been established in asthmatic patients targeting mitochondrial membrane domain containing 1 (TIMMDC1) and IL-6, respectively. In addition to asthma and COPD, lncRNAs also regulate CF lung disease. For example, McKiernan et al. (McKiernan et al., 2014) found that X inactivation-specific transcript (XIST) and Malat are differentially expressed in bronchial brushings of CF patients. Balloy et al. (Balloy et al., 2017) also found that several lncRNAs, such as maternally expressed gene 9 (MEG9) and bladder cancer-associated transcript 1 (BLACAT1), are downregulated in *Pseudomonas aeruginosa* (PA)-infected CF bronchial epithelial cells. In addition, lncRNA BGas was shown to be associated with CF by directly targeting and regulating CFTR (Saayman et al., 2016). Unfortunately, current studies on lncRNAs in bronchiectasis are limited and need further investigation. Collectively, these studies suggest that miRNAs and lncRNAs play a significant role in the pathogenesis and development of chronic inflammatory airway diseases.

## LncRNA–miRNA Interaction in Chronic Inflammatory Airway Diseases

**FIGURE 1:**
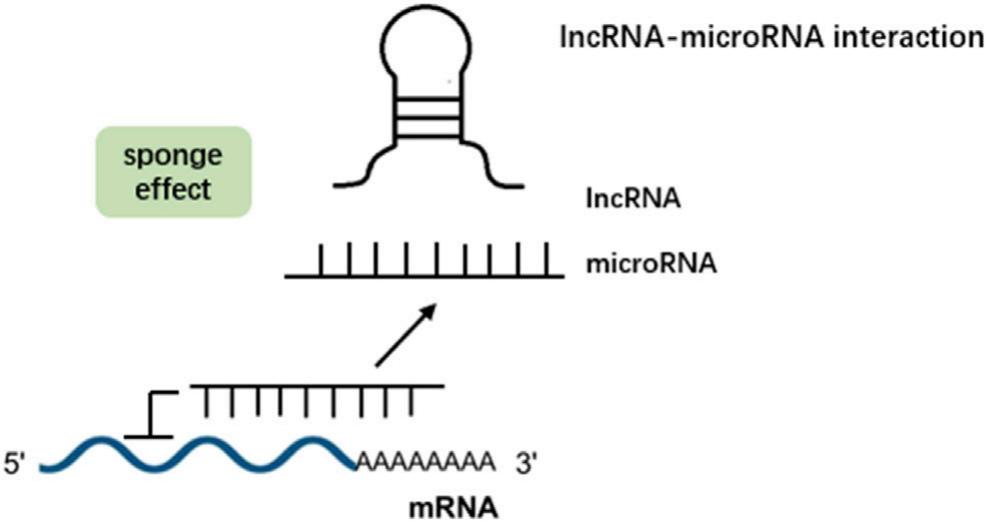
LncRNAs can act as a sponge for microRNAs. By binding to these microRNAs, they prevent microRNAs from binding to their target mRNAs, thereby abolishing post-transcriptional regulation.

In addition to the regulation where both miRNAs and lncRNAs act alone on mRNAs, accumulating evidence has demonstrated that lncRNAs can interact with miRNAs to regulate mRNA expression via various posttranscriptional mechanisms. Four types of lncRNA–miRNA interactions have been demonstrated: miRNA-triggered lncRNA decay, lncRNA generating miRNA, lncRNA–miRNA competition for mRNA, and lncRNA acting as miRNA sponges/decoys (Yoon et al., 2014). Mounting studies have shown that the latter is the most common mechanism involved in various diseases, such as cancer (López-Urrutia et al., 2019), cardiovascular diseases (Huang, 2018), rheumatoid arthritis (Han et al., 2022), and cerebral diseases (Mehta et al., 2021). Under such an interaction, lncRNAs prevent miRNAs from hindering the expression of target genes, such as mRNAs, and ultimately affect the function of cells by enhancing target mRNA expression (Li et al., 2018) (Figure 1). Recently, lncRNAs acting as miRNA sponges have been gradually revealed to be involved in chronic inflammatory airway diseases, such as COPD and asthma. PubMed was searched using the query “lncRNAs” AND “miRNAs” AND “COPD OR asthma OR bronchiectasis OR cystic fibrosis.” Articles regarding lncRNA-miRNA crosstalk discovery in chronic inflammatory airway diseases as well as *in vitro* and *in vivo* functional studies were included. They participate in the regulation of the proliferation, apoptosis, invasion, CD4^+^ T-cell differentiation, epithelial–mesenchymal transition (EMT), and inflammation of multiple relevant cell types in chronic inflammatory airway diseases. In the following sections, we describe the emerging roles and mechanistic functions of lncRNA–miRNA interactions in various cell types involved in chronic inflammatory airway diseases. Table 1 summarizes the crosstalk of lncRNA-miRNA-mRNA in COPD and asthma.

**TABLE 1:** The crosstalk of lncRNA-miRNA-mRNA in COPD and asthma.

Diseases	Sample type	Detecting technology	Identified lncRNAs	Sponge miRNAs	Target mRNAs	Function	Study
COPD	Human serum from COPD patients	qRT- PCR	LUCAT1	miR-181a-5p	Wnt/β- catenin	Promoted CSE-induced 16HBE cells apoptosis and inhibited proliferation	Zhao et al. (Zhao et al., 2021)
	Human peripheral blood from COPD patients	qRT-PCR	MEG3	miR-149-3p	NF-κB	Promoted CSE-induced 16HBE cells apoptosis	Lei et al. (Lei et al., 2021)
	Cell model	Microarray	RP11-86H7.1	miR-9-5p	NF-κB	Promoted TRAPM2.5-induced 16HBE cells inflammation	Zhao et al. (Zhao et al., 2020)
	Mice model	qRT-PCR	MIAT	miR-29c-3p	HIF3A	Promoted CSE-induced murine lung epithelial cells and fibroblasts apoptosis, inflammation, and EMT	Gu et al. (Gu et al., 2022)
	Human venous blood from COPD patients	qRT-PCR	OIP5-AS1	miR-410-3p	IL-13	Promoted CSE-induced 16HBE cells apoptosis and inflammation	Hao et al. (Hao et al., 2021)
	Human lung tissues from COPD patients	qRT-PCR	NNT-AS1	miR-582-5p	FBXO11	Promoted CSE-induced 16HBE cells apoptosis, inflammation, and airway remodeling	Mei et al. (Mei et al., 2020)
	Human lung tissues from COPD patients	qRT-PCR	XIST	miR-200c-3p	EGR3	Promoted CSE-induced 16HBE cells apoptosis and inflammation	Chen et al. (Chen et al., 2021a)
	Human induced-sputum and lung tissues from COPD patients	qRT-PCR	TUG1	miR-145-5p	DUSP6	Promoted CSE‐induced 16HBE cells and lung fibroblasts inflammation and airway remodeling	Gu et al. (Gu et al., 2019)
	Human lung tissues from COPD patients	qrt-PCR	SNHG5	miR-132	PTEN	Inhibited CSE-induced 16HBE cells apoptosis and inflammation	Shen et al. (Shen et al., 2020)
	Human serum from COPD patients	qrt-PCR	CASC2	miR-18a-5p	IGF1	Inhibited 16HBE cells apoptosis and inflammation	Liu et al. (Liu et al., 2021a)
	Human lung tissues from COPD patients	qrt-PCR	LOC729178	miR-144-3p	PHLPP2	Inhibited CSE-induced 16HBE cells inflammation	Wang et al. (Wang et al., 2021b)
	Human lung tissues from COPD patients	qrt-PCR	LINC00612	miR-31-5p	Notch1	Inhibited CSE-induced HPMECs apoptosis, inflammation, and oxidative stress	Luo et al. (Luo et al., 2020)
	Human lung tissues from COPD patients	qrt-PCR	MIR155HG	miR-128-5p	BRD4	Promoted CSE-induced HPMECs apoptosis and inflammation	Song et al. (Song et al., 2020)
	cell model	qrt-PCR	TUG1	miR-9a-5p	BCL2L11	Promoted CSE-induced HPMECs apoptosis	Chen et al. (Chen et al., 2021b)
Asthma	cell model	qrt-PCR	OIP5-AS1	miR-143-3p	HMGB1	Promoted Der p1-induced BEAS-2B cells inflammation and apoptosis	Cai et al. (Cai et al., 2020)
	Human peripheral blood CD4 + T cells from asthma patients	Microarray	MEG3	miR-17	RORγt	Inhibited Treg/Th17 ratio of CD4 + T cells in asthma patients	Qiu et al. (Qiu et al., 2019)
	Human peripheral blood mononuclear cells from asthmatic children	RNA- sequencing	TUG1	miR-29c	B7-H3	Promoted Th2 Cell differentiation	Sun et al. (Sun et al., 2021)
	Human serum from asthma patients	qrt-PCR	Malat1	miR-155	CTLA4	Regulated Th1/Th2 balance within CD4^+^ T cells	Liang et al. (Liang and Tang, 2020)
	Mice model	Western blot	PVT1	miR-15a-5p	PI3K/Akt/Mtor	Promoted Th1/Th2 imbalance of CD4+T cells	Wei et al. (Wei et al., 2020)
	Rat model	qrt-PCR	GAS5	miR-10a	BDNF	Promoted PDGF-BB-induced ASMCs proliferation and increased airway hyperresponsiveness	Zhang et al. (Zhang et al., 2018b)
	Rat model	qrt-PCR	Malat1	miR-133a	RyR2	Promoted B/TSMCs apoptosis and inflammation	Yang et al. (Yang and Wang, 2021)
	Cell model	qrt-PCR	NEAT1	miR-9-5p	SLC26A2	Promoted PDGF-induced ASMCs proliferation, migration, and inflammation	Wang et al. (Wang et al., 2021d)
	Rat model	qrt-PCR	TUG1	miR-138-5p	E2F3	Promoted PDGF-BB-induced ASMCs viability and migration	Zhou et al. (Zhou et al., 2021)
	Rat model	qrt-PCR	TUG1	miR-590-5p	FGF1	Promoted the ASMCs proliferation and migration	Lin et al. (Lin et al., 2019a)
	Human whole blood from childhood asthma patients	qrt-PCR	TUG1	miR-216a-3p	SMURF2	Promoted PDGF-BB-induced ASMCs proliferation and migration	Wang et al. (Wang and Chen, 2021)
	Mice model	qrt-PCR	TUG1	miR-181b	HMGB1	Promoted ASMCs proliferation and migration	Huang et al. (Huang et al., 2021)
	Human primary ASMCs from patients who underwent lung resection	qrt-PCR	Malat1	miR-150	Eif4E/Akt	Promoted PDGF-BB-induced ASMCs proliferation and migration	Lin et al. (Lin et al., 2019b)
	ASMCs isolated from bronchial biopsies from severe asthmatic subjects	qrt-PCR	CASC7	miR-21	PTEN/PI3K/Akt	Promoted sensitivity of ASMCs to corticosteroids	Liu et al. (Liu et al., 2020)
	Human serum from asthmatic children	qrt-PCR	H19	miR-21	PTEN/Akt	Inhibited PDGF-BB-induced ASMCs proliferation and migration	Yu et al. (Yu et al., 2021)
	Rat model	qrt-PCR	LINC-PINT	miR-26a-5p	PTEN	Inhibited PDGF-BB-induced ASMCs viability, migration, and MMP-1 and MMP-9 protein expression	Gao et al. (Gao et al., 2021)
	Cell model	qrt-PCR	RP5-857K21.7	miR-508-3p	PI3K/Akt/Mtor	Inhibited PDGF-BB-induced ASMCs proliferation, migration	Wang et al. (Wang et al., 2022)
	Mice model	Western blot	PVT1	miR-29c-3p	PI3K/Akt/Mtor	Promoted ASMCs proliferation and migration	Wei et al. (Wei et al., 2020)
	Cell model	qrt-PCR	FTX	miR-590-5p	JAK2	Promoted PDGF-BB-induced ASMCs proliferation and migration	Shen et al. (Shen et al., 2021)
	Cell model	qrt-PCR	LINC00882	miR-3619-5p	β-catenin	Promoted PDGF-BB-induced ASMCs proliferation	Liu et al. (Liu et al., 2019)

### LncRNAs Acting as miRNA Sponges in Bronchial Epithelial Cells

Bronchial epithelial cells are the first line of defence against environmental stimuli and play a critical role in maintaining normal airway function. Dysfunction of bronchial epithelial cells results in immune homeostasis disorder, persistent inflammation, oxidative stress, and EMT (Gao et al., 2015). The involvement of lncRNA sponging miRNA in the regulation of bronchial epithelial cells in chronic inflammatory airway diseases has been established by several studies. A number of lncRNAs including myocardial-infarction‐associated transcript (MIAT) (Gu et al., 2022), nicotinamide nucleotide transhydrogenase-antisense RNA1 (NNT-AS1) (Mei et al., 2020), XIST (Chen et al., 2021), and TUG1 (Gu et al., 2019) are upregulated in CSE-induced human bronchial epithelial cell line (16HBE) and interact with the miR-29c-3p/HIF3A axis, the miR-582-5p/FBXO11 axis, the miR-200c-3p/early growth response 3 (EGR3) axis, and the miR-145-5p/dual-specificity phosphatase 6 (DUSP6), respectively, to participate in CSE-induced 16HBE cell apoptosis and inflammation. Zhao et al. (Zhao et al., 2021) detected the expression of lncRNAs in the serum from patients with COPD and found that the expression of lung cancer-associated transcript 1 (LUCAT1) was significantly increased, especially in the smoker COPD group, and LUCAT1 levels were positively correlated with inflammatory cytokine (IL-1β, IL-6, and TNF-α) expression in COPD patients. Further studies in CSE-treated 16HBE cells showed that LUCAT1 acts as a miR-181a-5p sponge modulator of the expression of the downstream target genes TCF4, cyclin D1, c-Myc, and β-catenin. Silencing LUCAT1 alleviated the effects of CSE on 16HBE cell proliferation and apoptosis through the miR-181a-5p/Wnt/β-catenin pathway. The roles of Wnt/β-catenin signalling activation in cell proliferation, apoptosis, inflammation and injury repair (Zemans et al., 2011; Tanjore et al., 2013; Jang et al., 2017), as well as the potential in attenuating COPD pathogenesis, have been well demonstrated (Uhl et al., 2015). Furthermore, LUCAT1 could differentiate COPD patients from smokers and nonsmokers with high sensitivity, specificity, and accuracy. These findings suggest that LUCAT1/miR-181a-5p/Wnt/β-catenin axis may be a promising target for COPD treatment and that LUCAT1 may be a valuable indicator for differentiating COPD.

Lei et al. (Lei et al., 2021) reported that in the peripheral blood samples of smokers with COPD, the expression levels of lncRNA MEG3 and miR-149-3p were negatively correlated. In the subsequent COPD model induced by CSE *in vitro*, they found that MEG3 expression was significantly upregulated. The knockdown of MEG3 could directly target miR-149-3p to inhibit the activation of the NF-κB signalling pathway and promote proliferation while inhibiting apoptosis of CSE-treated HBE cells, suggesting that MEG3/miR-149-3p/NF-κB axis has a crucial function in the pathogenesis of COPD. In another report from traffic-related air pollution particulate matter 2.5 (TRAPM2.5)-induced 16HBE cells, significantly elevated lncRNA RP11-86H7.1 accompanied by activation of the NF-κB signalling pathway was detected (Zhao et al., 2020). The overexpressed RP11-86H7.1 could directly sponge miR-9-5p, enhance the expression levels of NFKB1, IL-6, IL-8, and tumor necrosis factor (TNF)-α, and promote the inflammatory response in TRAPM2.5-treated 16HBE cells, providing further insights into lncRNA–miRNA interactions regarding new treatment strategies for airway inflammatory diseases caused by PM2.5 exposure, such as COPD (Zhao et al., 2020). The two aforementioned studies showed that the NF-κB signalling pathway regulated by lncRNA–miRNA plays a crucial role in the cell inflammatory response, proliferation and apoptosis.

Hao and his colleagues (Hao et al., 2021) revealed that the levels of opa interacting protein 5-antisense RNA 1 (OIP5-AS1) were elevated in smokers and smokers with COPD, and there was a negative connection between OIP5-AS1 and FEV_1_. Furthermore, OIP5-AS1 showed a talent in differentiating smokers with COPD from smokers, as revealed by the area under the curve (AUC) of 0.903. Knockdown of OIP5-AS1 reversed the effects of CSE on 16HBE cell viability and apoptosis via miR-410-3p and IL-13. All of these findings indicated that OIP5-AS1 might serve as a diagnostic biomarker as well as a promising target for COPD treatment. In addition to a regulatory role in COPD, Cai et al. (Cai et al., 2020) found that OIP5-AS1 expression levels increased in an *in vitro* asthma model (Der p1-induced BEAS-2B cells) and that OIP5-AS1, serving as the molecular sponge of miR-143-3p, promoted Der p1-induced BEAS-2B cell apoptosis and inflammation by modulating high mobility group box-1 (HMGB1), a highly conserved ubiquitous protein that has been demonstrated to be associated with the development of asthma by promoting allergen-induced airway remodelling (Hou et al., 2015). These results provide a reference for OIP5-AS1 sponging different miRNAs as therapeutic targets in asthma and COPD.

In contrast to the aforementioned lncRNAs, three reported lncRNAs showed their abilities to suppress CSE-induced 16HBE cell apoptosis and inflammation by sponging miRNAs and affecting their target genes. For example, Shen et al. (Shen et al., 2020) reported that small nucleolar RNA host gene 5 (SNHG5) expression was significantly decreased in COPD tissues and that high SNHG5 expression was positively correlated with FEV_1_% in patients. In CSE-treated 16HBE cells, SNHG5 expression was reduced, and miR-132 was detected as an SNHG5 miRNA target. Simultaneously, PTEN was confirmed to serve as a target gene of miR-132. SNHG5 upregulation reversed the effects of CSE on 16HBE cell proliferation, apoptosis, and inflammatory cytokine levels (IL-1β, IL-6 and TNF-α) by targeting the miR-132/PTEN axis. As described by a previous study, PTEN plays a crucial role in regulating the inflammatory response in COPD (Yanagisawa et al., 2017). These data suggest that SNHG5/miR-132/PTEN is involved in COPD progression and might serve as a potential prognostic biomarker. The other two lncRNAs, CASC2 and LOC729178, also act as miRNA sponges in COPD; their overexpression alleviated CSE-induced 16HBE cell inflammatory injury and apoptosis via the CASC2/miR-18a-5p/insuline-like growth factor I (IGF1) and LOC729178/miR-144-3p/PH domain leucine-rich repeat protein phosphatase 2 (PHLPP2) axes, respectively (Liu et al., 2021a; Wang et al., 2021b). In addition, the level of CASC2 expression was shown to be positively associated with FEV_1_ (Liu et al., 2021a), which might be a promising biomarker for disease diagnosis and to have diagnostic accuracy in distinguishing COPD patients from smokers.

The abnormal airway epithelium contributes to both CF and bronchiectasis (Peng et al., 2020; Chen et al., 2018; Barbry et al., 2020). Insufficient growth and differentiation of epithelial cells can lead to persistent mucosal injury that promotes bacterial colonization and neutrophilic inflammation. There has been evidence that the lncRNAs MEG9 and BLACAT1 are downregulated in CF bronchial epithelial cells infected with PA (Balloy et al., 2017). MiR-145-5p and miR-494 are directly involved in the dysregulation of CFTR in bronchial epithelial cells, as mentioned above (Oglesby et al., 2013). Further research is needed to investigate whether the lncRNA-miRNA-mRNA axis affects bronchial epithelial cells in CF and bronchiectasis, which may present novel therapeutic opportunities.

### LncRNAs Act as miRNA Sponges in Pulmonary Microvascular Endothelial Cells

Angiogenesis and microvascular remodelling are known features of chronic inflammatory airway diseases, such as COPD and asthma (Alagappan et al., 2013; Bakakos et al., 2016). Cigarette smoking can initiate pulmonary vascular impairment by directly injuring endothelial cells, resulting in the enhancement of cell apoptosis and vascular permeability, reduced epithelial barrier formation, angiogenesis and high levels of oxidative stress, thereby contributing to COPD development (Alagappan et al., 2013; Long et al., 2018). A number of lncRNAs have been thought to be promising for the treatment of COPD by regulating pulmonary microvascular endothelial cell function through targeting the miRNA/mRNA axis. A recent report demonstrated the downregulation of LINC00612 in COPD tissues and in human pulmonary microvascular endothelial cells (HPMECs) exposed to CSE (Luo et al., 2020). The upregulation of LINC00612-inhibited cell apoptosis, inflammation, and oxidative stress in HPMECs induced by CSE positively regulated Notch signalling by sponging miR-31-5p, indicating that LINC00612/miR-31-5p/Notch axis could function as a potential biomarker and treatment target for COPD in the future. Interestingly, the Notch signalling pathway has been acknowledged to play a significant role in asthma (Kang et al., 2009; Gu et al., 2012). Therefore, whether LINC00612/miR-31-5p/Notch can be also a therapeutic target for asthma treatment needs to be explored in the future. Song et al. (Song et al., 2020) found increased expression of MIR155 host gene (MIR155HG) in lung tissues of smokers with COPD compared with controls with COPD and CSE-treated HPMECs. In CSE-treated HPMECs, MIR155HG acts as a sponge for miR-128-5p, and luciferase reporter assays subsequently showed an interaction between miR-128-5p and BRD4. BRD4 has been revealed to play a vital role in the regulation of inflammation, oxidative stress, and innate immunity in COPD patients (Devaiah et al., 2016; Malhotra et al., 2017; Tang et al., 2019). Elevated MIR155HG expression promoted HPMEC apoptosis and inflammation *in vitro* through the miR-128-5p/BRD4 regulatory axis. MIR155HG deletion using small interfering RNAs (siRNAs) *in vitro* suppressed apoptosis and inflammation in CSE-treated HPMECs, indicating therapeutic potential (Fehrenbach et al., 2017). In addition, Li et al. (Li et al., 2019b) found that MIR155HG regulated M1/M2 macrophage polarization in COPD by regulating IL-1β, IL-10, IL-12, and TNF-α expression, suggesting that MIR155HG might be involved in the development of COPD. Therefore, more studies are needed to confirm whether MIR155HG can regulate macrophage polarization by sponging miRNA, providing a novel treatment strategy for COPD. Chen et al. (Chen et al., 2021b) investigated the function of TUG1 in an *in vitro* COPD model with CSE-treated HPMECs and showed that overexpression led to apoptosis in HPMECs by modulating the miR-9a-5p/Bcl-2-like protein 11 (BCL2L11) axis, suggesting that TUG1/miR-9a-5p/BCL2L11 is a potential effective target for COPD treatment.

Despite the crucial role of angiogenesis and microvascular remodelling in asthma, studies on the role of lncRNA-miRNA crosstalk in the regulation of HPMECs function contributing to the development of asthma are rare, which requires further exploration.

### LncRNAs Acting as miRNA Sponges in T Lymphocytes

Lymphocytes are the main immune cells mediating airway inflammation in COPD and asthma. T lymphocytes have been shown to play a central role in the pathophysiology of asthma. T lymphocytes are generally divided into two major types: CD4+T cells and CD8+T cells. CD4^+^ T cells initiate inflammatory and allergic responses in allergic asthma, leading to two asthma phenotypes: Th2 and non-Th2 (Sze et al., 2020). Th2-asthma (i.e., eosinophilic asthma) is well established to play a leading role in asthma development as more than half of asthma cases have a Th2 phenotype, characterized by the secretion of high levels of IL-4, IL-5, and IL-13 by Th2 cells (Masuda et al., 2008; Hammad and Lambrecht, 2021). Non-Th2 asthma (i.e., neutrophilic asthma), by contrast, is characterized by the secretion of high levels of interferon-gamma (IFN-γ) and IL-17 by Th1 and Th17 cells (Sze et al., 2020). Biased differentiation of CD4^+^ T cells contributes to abnormal inflammation in asthma (Wu et al., 2016). LncRNAs and miRNAs alone have been shown to play regulatory roles in asthma by regulating CD4^+^ T-cell function (Wang et al., 2021c). Notably, three studies observed a novel regulatory role of lncRNAs targeting miRNAs in regulating the Th1/Th2 balance (Liang and Tang, 2020; Wei et al., 2020; Sun et al., 2021). For example, Sun et al. (Sun et al., 2021) observed that TUG1 is differentially expressed in the monocytes of asthmatic children. In further *in vitro* experiments on macrophages treated with house dust mites, they demonstrated that TUG1 could regulate B7-H3 expression in macrophages by sponging miR-29c, which then controls Th2 differentiation, providing novel potential diagnostic biomarkers and therapeutic targets for asthma. Liang et al. (Liang and Tang, 2020) demonstrated that MALAT1 could sponge miR-155, affecting the Th1/Th2 balance through a cytotoxic T-lymphocyte-associated antigen 4 (CTLA-4)-dependent mechanism, which might aid in the development of therapies for ameliorating inflammation in asthma. Wei et al. (Wei et al., 2020) also investigated the function of PVT1, showing that overexpression significantly decreased the Th1/Th2 ratio in CD4^+^ T cells by depressing miR-15a-5p expression and therefore induced PI3K–Akt–mTOR signalling. Previous studies found that PI3K/Akt activation facilitated the differentiation of Th cells into Th2-like CD4^+^ T cells (Ward and Peter, 2003; Arimura et al., 2004). Therefore, the lncRNA PVT1/miR-15a-5p/PI3K-Akt-mTOR axis was implicated in asthma development by promoting Th1/Th2 imbalance.

In addition to the aforementioned regulatory role of the lncRNA–miRNA axis in Th1/Th2 imbalance, Qiu et al. (Qiu et al., 2019) found that the lncRNA MEG3 plays a role in the regulatory T-cell (Treg)/Th17 balance by sponging target miRNAs. They found that MEG3 expression levels were upregulated and negatively associated with miRNA-17 expression in CD4^+^ T cells of patients with severe asthma. Silencing of MEG3 expression using siRNA in CD4^+^ T cells reduced the expression of the Th17 transcription factors orphan receptor (ROR)γt and IL-17. Additionally, miR-17 was detected as a target of MEG3, and it could suppress the Th17 response or promote it by targeting RORyt, suggesting that MEG3/miRNA-17/RORyt axis plays a role in the Th17 imbalance in asthma. Previous studies found that the frequency of Th17 cells was markedly increased in patients with moderate to severe asthma and that Treg/Th17 imbalance is more closely related to asthma progression and severity than Th1/Th2 imbalance (Shi et al., 2011; Wang et al., 2018b). Therefore, we can assume that MEG3/miRNA-17/RORyt axis could be a biomarker of asthma severity. However, Feng et al. (Feng et al., 2020) found that MEG3 was downregulated in the serum of patients with neutrophilic asthma compared with controls, which contradicts the above finding and requires further research and validation.

Although the critical role of CD4+T cells in the physiopathology is indisputable, recent evidence suggests that CD8+T cells may also be involved in asthma. Den et al. (den Otter et al., 2016) found that the decline of FEV_1_ both at baseline and follow-up correlated with the number of CD8+T cells in asthmatic airways, hinting a positive correlation between the disease severity and CD8+T cells. Tsitsiou et al. (Tsitsiou et al., 2012), using transcriptome analysis, showed that severe asthma is associated with the activation of circulating CD8^+^ T cells but not CD4^+^ T cells, which is correlated with the changes in the expression of miR-146a/b and miR-28-5p as well as 167 different lncRNAs that might regulate CD8^+^ T-cell function. Therefore, the regulatory roles of lncRNAs or miRNAs or lncRNA–miRNA interactions in CD8^+^ T-cell function merit further exploration. If possible, this might provide a novel therapeutic approach to the treatment of severe asthma.

As with asthma, Th17-mediated pulmonary inflammation is also crucial to COPD (Ponce-Gallegos et al., 2017). In a COPD mouse model, He et al. (He et al., 2021) reported that upregulated miRNA-21 could promote Th17 cell differentiation, contributing to increased levels of inflammatory cytokines via the Smad7/TGF-β pathway. However, the role of lncRNA-miRNA-mRNA axis in COPD through regulating T cells has rarely been studied, which needs to be investigated.

### LncRNAs Act as miRNA Sponges in Airway Smooth Muscle Cells

Airway remodelling is an important feature of chronic inflammatory airway disease, especially COPD and asthma. An important contribution to airway remodelling and irreversible bronchoconstriction is increased airway smooth muscle (ASM) mass, which is thought to be brought on by airway smooth muscle cell (ASMC) proliferation, migration and hypertrophy (Salter et al., 2017). The role of lncRNA–miRNA–mRNA regulatory network in the asthmatic process by modulating platelet-derived growth factor (PDGF)-BB-stimulated ASMCs has been increasingly reported in recent years. For example, lncRNA growth arrest-specific transcript 5 (GAS5), lncRNA nuclear paraspeckle assembly transcript 1 (NEAT1), and lncRNA PVT1 can act as “sponges” of miR-10a, miR-9-5p, and miR-29c-3p, respectively, and are involved in the biological processes of ASMC proliferation, migration, apoptosis and inflammation in asthmatic patients (Zhang et al., 2018b; Wei et al., 2020; Wang et al., 2021d). TUG1, serving as the molecular sponge of miR-138-5p, miR-590-5p, miR-216a-3p, and miR-181b, has been confirmed to be involved in ASMC proliferation and migration in human asthma by modulating E2F transcription factor 3 (E2F3), fibroblast growth factor 1 (FGF1), Smurf2 and HMGB1, respectively (Lin et al., 2019a; Huang et al., 2021; Wang and Chen, 2021; Zhou et al., 2021). Similarly, the lncRNA Malat1 also contains multiple binding sites to miRNAs and sponges miR-150 (Lin et al., 2019b) and miR-133a (Yang and Wang, 2021) in addition to the aforementioned miR-155 (Liang and Tang, 2020), thereby regulating PDGF-BB-stimulated ASMC proliferation and migration. These findings provide a theoretical basis for lncRNAs-miRNAs-mRNAs axis being potential therapeutic targets for asthma.

Multiple signalling pathways are involved in the pathogenesis of asthma by regulating airway smooth muscle cell function. Liu et al. (Liu et al., 2020) found that lncRNA CASC7 inhibits the PI3K/Akt signalling pathway by targeting miR-21 and promotes the phosphorylation of glucocorticoid receptors to increase the sensitivity of glucocorticoids in patients with severe asthma. Indeed, miR-21 overexpression could lead to steroid-insensitive airway inflammation and airway hyperresponsiveness (AHR) (Kim et al., 2017). These findings indicated the therapeutic potential of CASC7/miR-21/PI3K/Akt axis in AHR. Yu et al. discovered that lncRNA H19 overexpression reduced the growth and migration of ASMCs by acting on the miR-21/PTEN/Akt pathway (Yu et al., 2021). Interestingly, several studies with an asthmatic mouse model and in asthmatic children indicate that the upregulation of miR-21 promotes increased expression of Th2 cytokines and inhibits Th1 cytokine expression (Lu et al., 2009; Elbehidy et al., 2016; Hammad Mahmoud Hammad et al., 2018). Therefore, lncRNA CASC7/miR-21/PI3K/Akt axis and H19/miR-21/PTEN/Akt axis are potential therapeutic targets for asthma, and whether they can regulate T-cell differentiation to treat asthma needs further study. Gao et al. (Gao et al., 2021) found that LINC p53-induced transcript (PINT) overexpression decreased the serum levels of IgE and AHR, airway inflammation, and pathological changes in the lung in an asthma rat model. *In vitro*, LINC-PINT overexpression retarded abnormal ASMC growth to attenuate the progression of asthma by regulating the miR-26a-5p/PTEN axis. PTEN is a phosphatase that acts as a negative regulator of PI3K/AKT signalling, and the PTEN/PI3K/AKT signalling pathway plays multifaceted roles in asthma pathogenesis (Yoo et al., 2017). Furthermore, the PI3K/AKT/mTOR pathway was also involved in the regulation of AMSC proliferation and migration by lncRNA RP5-857K21.7 sponging miR-508-3p (Wang et al., 2022).

In addition to the PI3K/AKT/mTOR pathway, the janus kinase (JAK) signalling pathway has been shown to play a key role in asthma (Li and Wang, 2018; KimHye et al., 2021). Shen et al. (Shen et al., 2021) showed that lncRNA FTX could inhibit the proliferation and migration of ASMCs caused by PDGF-BB by targeting miR-590-5p and that JAK2 was a direct target of the FTX/miR-590-5p signalling axis, the overexpression of which reversed the inhibitory effect on proliferation and migration and the apoptosis-inducing effect of miR-590-5p in ASMCs, highlighting the crucial regulatory role of the FTX/miR-590-5p/JAK2 axis in ASMC proliferation, migration, and apoptosis.

Liu et al. (Liu et al., 2019) demonstrated that LNC00882 promotes the proliferation of ASMCs by enhancing Wnt/β-catenin signalling by sponging microRNA-3619-5p, supporting a role of LNC00882/microRNA-3619-5p/Wnt/β-catenin in airway remodelling. Another study from Perry et al. (Perry et al., 2014) found that LINC00882 is complementary for miR-371 (a total of 7 sites) through microarray and quantitative real-time PCR. MiR-371 has been confirmed to have the ability to inhibit Runt-related transcription factor 3 (Runx3), a transcription factor that promotes Th1 differentiation, by combining the other four miRNAs, thereby modulating the Th1/Th2 balance in asthma (Qiu et al., 2017). Whether LNC00882 modulates the Th1/Th2 balance by binding to miR-371 remains to be investigated.

Airway smooth muscle cells act as the main effector cells and their proliferation represents a major characteristic of airway remodelling in COPD. However, few studies explore the role of lncRNA–miRNA–mRNA axis in COPD by regulating ASMCs function, which needs to be confirmed further.

### LncRNAs Act as miRNA Sponges in Other Pulmonary Cell Types

The key features of chronic inflammatory airway disease pathophysiology are small airway fibrosis, structural damage, and extracellular matrix deposition resulting in remodelling of the airways (Ma et al., 2020; Mehrad et al., 2012; Rout-Pitt et al., 2018). Airway remodelling in patients with these diseases is related to EMT, in which fibroblasts and airway epithelial cells are the two central effector cells involved (Guan et al., 2020; Sohal et al., 2010). It has been shown that the MIAT/miR-29c-3p/HIF3A and TUG1/miR-145-5p/DUSP6 axis are integral in promoting EMT and collagen deposition among lung fibroblast through upregulation of 3b1;-SMA, vimentin and collagen I levels (Gu et al., 2022). They also promote apoptosis and inflammation induced in CSE. Additionally, MIAT/miR-29c-3p/HIF3A appear to have a regulatory role in CSE-induced inflammation in the murine type II alveolar epithelial cell line MLE12 (Gu et al., 2022).

Furthermore, other immune cells, such as macrophages and neutrophils, also contribute to the inflammatory and immune process responsible for much of the pathology found in patients with chronic inflammatory airway disease. Previous studies have shown that ncRNAs act as crucial regulators in these cells. Shi (Shi et al., 2020), Li (Li et al., 2019), and Pei. et al. (Pei et al., 2020) found that ncRNAs such as AK085865, MIR155HG, miR-142-5p, and miR-130a-3p could affect macrophage polarization in the lungs, as well as related airway inflammation and airway remodelling in COPD and asthma. Tazi et al. (Tazi et al., 2016) found that elevated miRc1/miR17-92 cluster expression negatively regulated autophagy and CFTR function in CF macrophages. To date, only a few studies have explored the role of ncRNA in neutrophils. Neutrophil-derived miRNA-223-3p has been shown to modulate Toll-like receptors (TLRs)/Th17 and the endoplasmic reticulum stress response in asthmatic sputum (Gomez et al., 2020). Moreover, MiR-636 was found to increase the neutrophils of CF patients' blood and might serve as a new biomarker (Bardin et al., 2019). Currently, studies that explore the role of the lncRNA-miRNA-mRNA axis in these cells are lacking, and they ought to be encouraged to contribute to the development of novel therapeutic approaches for chronic inflammatory airway diseases.

## Clinical Implications in Chronic Inflammatory Airway Diseases

In recent years, a growing number of studies have demonstrated that crosstalk among lncRNAs, miRNAs and mRNAs is involved in the pathophysiology of chronic inflammatory airway diseases. Recent studies have focused on lncRNA-miRNA crosstalk as a possible biomarker of COPD and asthma. While the axis influences many biological processes in COPD, including diagnosis and treatment, research into asthma attempts to identify phenotypes and predict therapeutic responses.

### LncRNA–miRNA–mRNA Axis in COPD

**FIGURE 2:**
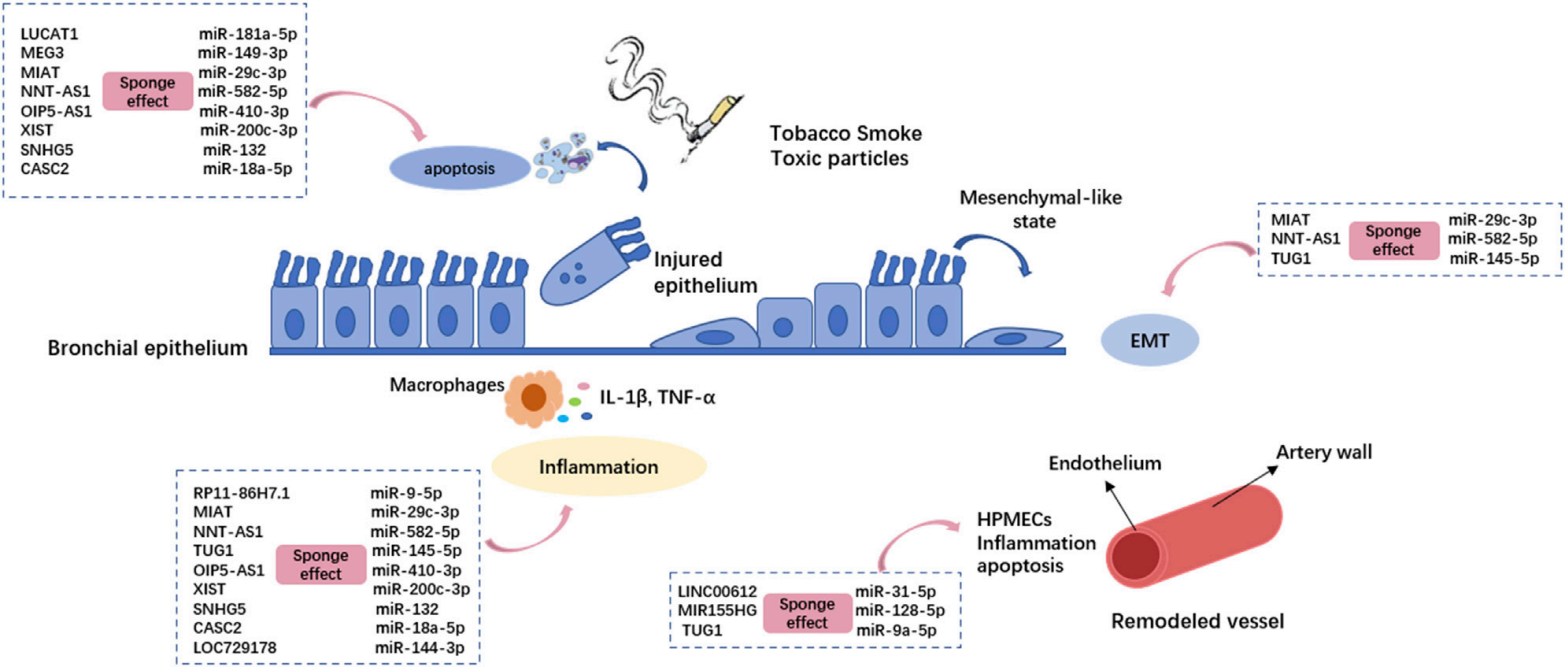
The lncRNA-miRNA crosstalk is involved in the pathogenesis of COPD by regulating bronchial epithelial cells and pulmonary microvascular endothelial cells. Different lncRNAs regulate different miRNAs through the sponge effect, and then inhibit Mrna. The dotted boxes in the picture show typical lncRNA-miRNA interactions that function in COPD, which correspond to the cellular process they act on through pink arrows. HPMECs: Human pulmonary microvascular endothelial cells; EMT: epithelial–mesenchymal transition.

Thirteen lncRNAs are implicated in the physio-pathological process of COPD by sponging miRNAs (Figure 2). Of these, eleven differentially expressed lncRNAs have been found in human bio-samples (including whole blood, serum, sputum, and lung tissue samples) from COPD patients: NNT-AS1 (Mei et al., 2020), MEG3 (Lei et al., 2021), LUCAT1 (Zhao et al., 2021), SNHG5 (Shen et al., 2020), CASC2 (Liu et al., 2021a), TUG1 (Gu et al., 2019), OIP5-AS1 (Hao et al., 2021), LOC729178 (Wang et al., 2021), XIST (Chen et al., 2021), LINC00612 (Luo et al., 2020), and MIR155HG (Song et al., 2020). There is a possibility that these lncRNAs could be clinically helpful in diagnosing and treating COPD. Compared to smokers without COPD, LUCAT1, CASC2, and OIP5-AS1 show a more sensitive expression pattern, thus enabling a helpful distinction between smokers with and without COPD. There is also evidence that LUCAT1 expression correlates with inflammation in COPD patients and that TUG1, SNHG5, CASC2, and OIP5-AS1 expression levels correlate with the severity of airflow limitation, supporting their roles as potential biomarkers for COPD progression.

Moreover, TUG1 may also be a novel and promising biomarker for COPD complicated with PAH. Wang et al. (Wang et al., 2019) previously demonstrated that TUG1 was significantly upregulated in patients with PAH and that TUG1 knockdown significantly prevented the development of PAH in vivo. This may provide multiple opportunities to further investigate a role of the lncRNA‐miRNA‐mRNA axis in COPD complicated with PAH.

Smoking and aberrant epithelial responses are risk factors for COPD and lung cancer (Murray et al., 2017). A large proportion of lung cancer patients showed a history of COPD, and the possible link between COPD and lung cancer has been demonstrated (Ytterstad et al., 2016; Hou et al., 2019). The blood levels of MEG3 are higher in COPD patients but lower in non-small-cell lung cancer patients (Zhao et al., 2019; Lv et al.,2021). This contradictory finding requires further research and validation.

### LncRNA–miRNA–mRNA Axis in Asthma

**FIGURE 3:**
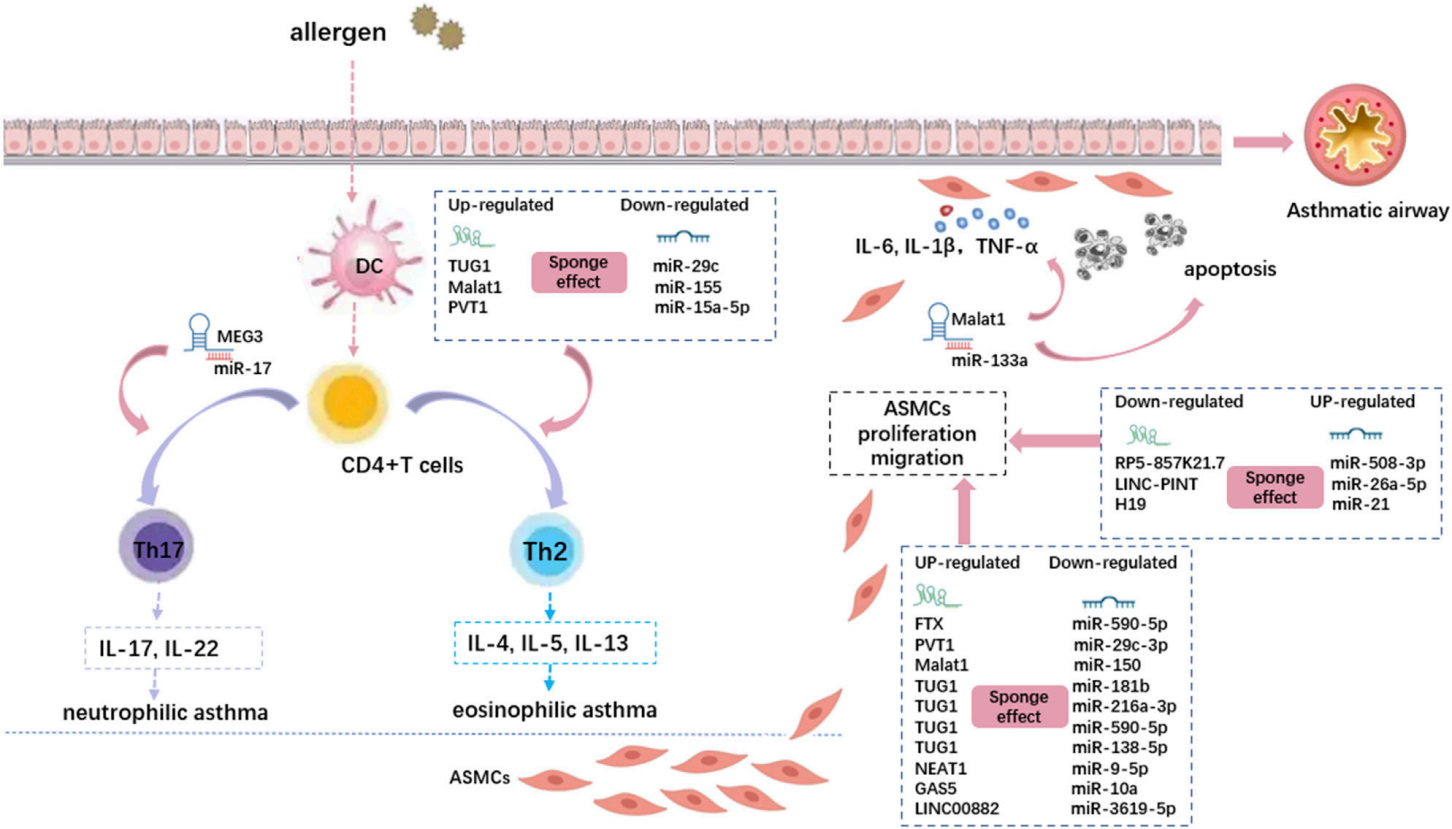
The lncRNA-miRNA crosstalk is involved in the pathogenesis of asthma by regulating CD4^+^ T cells and airway smooth muscle cells. Different lncRNAs regulate different miRNAs through the sponge effect, and then inhibit Mrna. The dotted boxes in the picture show typical lncRNA-miRNA interactions that function in asthma, which correspond to the cellular process they act on through pink arrows. DC: Dendritic cell; ASMCs: airway smooth muscle cells.

Thirteen lncRNAs sponging various miRNAs play critical roles in airway epithelial cells, smooth muscle cells, and T cells in asthma (Figure 3). MEG3 (Qiu et al., 2019), TUG1 (Sun et al., 2021), Malat1 (Liang and Tang, 2020), CASC7 (Liu et al., 2020), and H19 (Yu et al., 2021) showed differential expression in asthmatic patients. Various asthma phenotypes might be treated differently in terms of clinical management. For example, inhaled corticosteroids (ICS) are less effective in treating neutrophilic asthma, particularly in patients with severe ICS resistance (Heffler et al., 2018). A key component of precision medicine is the identification of accurate biomarkers that can be used to distinguish different disease phenotypes. These biomarkers can help determine the optimal treatment for each patient. Various lncRNA–miRNA–mRNA axes are associated with asthma severity or inflammatory phenotypes. TUG1 and Malat1 promote Th2 asthma through the TUG1/miR-29c/B7-H3 axis and Malat1/miR-155/CTLA4 axis, respectively, whereas MEG3 promotes non-Th2 asthma through the MEG3/miR-17/RORγt axis. In addition, the lncRNA CASC7/miR-21/PI3K/Akt axis may be beneficial for alleviating AHR in asthmatic patients by increasing sensitivity to glucocorticoids.

### LncRNA–miRNA–mRNA Axis in Other Chronic Inflammatory Airway Diseases

In addition to COPD and asthma, CF and bronchiectasis are usually defined as chronic inflammatory airway diseases. Chronic infection, inflammation, mucus hypersecretion, and bronchiectasis are their common features. As we described before, there is now growing literature to suggest that ncRNAs may be novel therapeutic targets for CF via the regulation of inflammation and mucus synthesis or directly/indirectly target CFTR. Notably, lncRNA MEG9 and lncRNA BLACAT1 were found to be significantly downregulated in PA-infected CF bronchial epithelial cells (Balloy et al., 2017). Interestingly, Huang et al. (Huang et al., 2019) found that differentially expressed miR-223-3p and miR-92b-5p were associated with PA colonization in bronchiectasis patients, and their expression levels were significantly correlated with sputum inflammatory biomarker levels (IL-β and IL-8), suggesting that these miRNAs could be biomarkers associated with PA colonization. Although current studies on the lncRNA–miRNA–mRNA axis in CF and bronchiectasis are limited, these findings provide clues for it being a therapeutic target to mitigate the adverse impacts caused by PA colonization in CF or bronchiectasis. Therefore, more investigations either *in vivo* or *in vitro* are urgently needed on this topic to better recognize the role of the lncRNA–miRNA–mRNA axis in chronic inflammatory airway diseases.

## Conclusion and Perspectives

In recent years, a growing number of studies confirm that lncRNAs and miRNAs have multifaceted and cell-type-specific functions, and might be involved in the pathogenesis and development of chronic inflammatory airway diseases, particularly COPD and asthma. This review sheds light on the expression profiles of lncRNA–miRNA–mRNA axis in various cell types involved in the pathophysiology of chronic inflammatory airway diseases and their potential function as diagnostic biomarkers and therapeutic targets for COPD and asthma via the regulation of cell proliferation, migration, differentiation, apoptosis, and inflammation.

Notably, the crosstalk of lncRNA and miRNA has excellent expectations regarding their potential as biomarkers for COPD diagnosis and complications, identifying asthma phenotypes and predicting therapeutic responses to glucocorticoids. Clinically, COPD and asthma are chronic airway inflammatory diseases with different pathogenesis but some overlaps. COPD and asthma involve airway inflammation and airway remodelling mediated by epithelial cells, T lymphocytes, endothelial cells, and airway smooth muscle cells. And several patients have both COPD and asthma features clinically. MEG3, OIP5-AS1, and TUG1 are involved in asthma and COPD pathogenesis by sponging different miRNAs. However, few studies have found that the lncRNA-miRNA-mRNA axis is associated with both COPD and asthma. Further research into ncRNA and ncRNA axis as biomarkers for asthma overlap COPD is warranted, which may contribute to asthma overlap COPD treatment.

CF and bronchiectasis are both characterized by chronic infection, mucus hypersecretion, and bronchiectasis. To date, the lncRNA-miRNA-mRNA axis has not been systematically studied concerning CF and bronchiectasis. Several ncRNAs, such as MEG9, BLACAT1, miRc1/miR17-92, miR-223-3p, miR-92b-5p and miR-636, have been found to be associated with airway epithelial cells, neutrophils, or macrophages in CF and bronchiectasis. Further research is required into the role of the lncRNA/miRNA/mRNA axis in CF and bronchiectasis.

There is still a long way to go to explore the functions and mechanisms of lncRNA–miRNA crosstalk. Firstly, although studies on the lncRNA-miRNA-mRNA axis have widely used mature detection technologies, including microarrays, RNA-sequencing, qRT-PCR, western blot, luciferase reporter assays, and RNA pull-down experiments, the reliability of detection results remains to be confirmed, as it may be affected by the technology types, sample preparation and storage, and the *in vitro* environment. Secondly, ncRNAs seem to be stable in human biofluids while some studies have raised concerns about sample quality, storage time and temperature that may affect the lncRNA-miRNA-mRNA network (Lorenzen and Thum, 2012; Jalali et al., 2013; Lorenzen et al., 2015; Weckbach et al., 2016; Glinge et al., 2017), which need further investigation. Thirdly, most published studies focus on the “sponge” effect between lncRNA and miRNA, the other lncRNA functionalities such as decoy, guide and scaffold affecting chronic inflammatory airway diseases need to be further studied, which may provide novel therapeutic implications. Finally, several challenges also need to be addressed before implementing lncRNA-miRNA-mRNA axes in clinical settings, including cell/tissue-specific delivery, binding affinity, stability, and sophisticated screening technologies.

In conclusion, there is no doubt that a better understanding of the crosstalk between lncRNAs and miRNAs will provide better strategies for disease diagnosis and treatment and that ncRNA networks will emerge as promising biomarkers and therapeutic targets of chronic inflammatory airway diseases, especially COPD and asthma in the near future.
